# Case report: Intracranial lesions of cat-scratch disease mimicking an atypical meningioma

**DOI:** 10.3389/fneur.2023.1080331

**Published:** 2023-02-08

**Authors:** Qiang Fang, Pengju Wang, Shanshan Qin, Shangxin Liu, Jingzhen He

**Affiliations:** ^1^Department of Radiology, Qilu Hospital of Shandong University, Jinan, China; ^2^Department of Medical Imaging, Binzhou Medical University, Binzhou, China

**Keywords:** cat-scratch disease (CSD), *Bartonella henselae*, magnetic resonance imaging, computed tomography, meningioma–pathology

## Abstract

**Objectives:**

Cat-scratch disease (CSD) is an infectious disease caused by *Bartonella henselae*. The most typical symptom of patients with CSD is regional lymphadenopathy, while central nervous system lesions related to CSD are rare. Here, we present a case of an aged woman with CSD involving the dura mater with a manifestation similar to that of an atypical meningioma.

**Methods:**

The patient was followed up by our neurosurgery and radiology teams. Clinical information was recorded, and the pre- and post-operation CT results and magnetic resonance imaging (MRI) changes were collected. The paraffin-embedded tissue was sampled for the polymerase chain reaction (PCR) test.

**Results:**

In this study, we present the details of a 54 year-old Chinese woman admitted to our hospital with a paroxysmal headache for 2 years that had worsened in the past 3 months. Brain CT and MRI showed a meningioma-like lesion below the occipital plate. En bloc resection of the sinus junction area was performed. A pathological examination showed granulation tissue and fibrosis with acute and chronic inflammation, granuloma, and central stellate microabscess, which was suspected as the cat-scratch disease. The paraffin-embedded tissue was sampled for a polymerase chain reaction (PCR) test to amplify the corresponding pathogen gene sequence, which was *Bartonella henselae*.

**Conclusion:**

The case in our study underscores the fact that the incubation period of CSD may be very long. On the contrary, CSD can involve the meninges, resulting in tumor-like lesions.

## Introduction

Cat-scratch disease (CSD) is an infectious disease caused by *Bartonella henselae*, a gram-negative bacillus, which is transmitted to humans through a scratch or bite from an infected cat. It is more prevalent in children and adolescents (1).

The clinical symptoms of most patients are skin eruption, regional lymphadenopathy, and low fever, most of which are self-limited (2). However, in rare cases of atypical CSD, the lesions can spread to organs like the liver, the bones, and the spleen (3). Some cases even involve the nervous system with the manifestation of encephalitis accompanied by fever, headache, decreased wakefulness, and epilepsy (4–6). Differential diagnosis of CSD can be challenging for both clinicians and radiologists as various manifestations of multiple systems. In this article, we present the case of a woman with CSD involving the dura mater with a manifestation similar to that of an intracranial tumor.

## Case description

**Figure 1:**
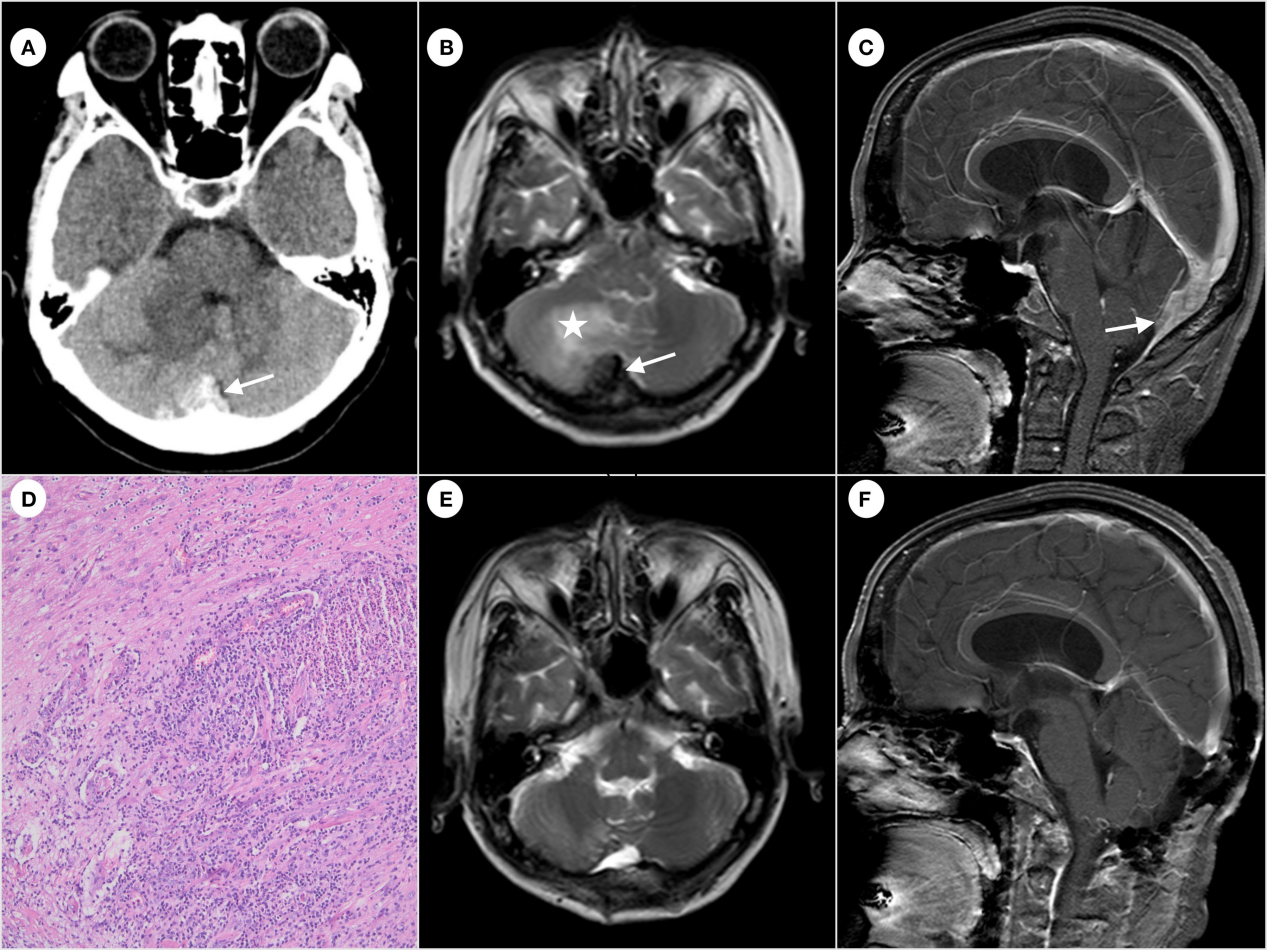
Computed tomography of the brain showing irregular high-density mass (arrow) below the occipital plate **(A)**. Hypointense (arrow) on T2-weighted magnetic resonance imaging and edema (star) observed near the right cerebellar hemisphere **(B)**. The enhanced scan shows significant enhancement and a dural tail sign (arrow) **(C)**. Formation of granuloma and central stellate microabscess. The image was acquired at 100× magnification **(D)**. The follow-up MRI shows the disappearance of the residual lesion in the surgical area **(E, F)**.

A 54 year-old woman was admitted to the Qilu Hospital of Shandong University for paroxysmal headache for 2 years that had worsened in the past 3 months without fever, nausea, and vomiting. The patient had no lymphadenopathy and denied exposure to fleas recently. The patient had normal muscle tone of limbs, negative pathological findings, normal results of routine blood tests, and negative tumor markers. The patient had no relevant past medical history, no medication history, and no travel history. In addition, the patient denied consuming undercooked meat. The CT scan showed an irregular high-density mass below the occipital plate, with patchy low-density areas in the right cerebellar hemisphere (Figure 1A), which the radiologist suspected to be a dural tumor and the neurosurgeon recommended performing MRI. The MRI scan showed that the lesion with a broad base was connected to the dura mater, with slightly shorter T1 and T2 signals (Figure 1B). The enhanced scan showed a significant enhancement and a dural tail sign (Figure 1C). The patchy area of no enhancement in the right cerebellar hemisphere was considered edema. The patient was diagnosed with atypical meningioma by radiologists and neurosurgeons before the operation. En bloc resection of the sinus junction area was performed, and then, the fascia lata of the left lower limb was used to repair the dura mater. While the lesion involving the venous sinus was not completely resected, the pathological examination showed granulation tissue and fibrosis with acute and chronic inflammation, granuloma, and central stellate micro-abscess (Figure 1D), which was suspected as the cat-scratch disease (CSD) by the pathologist. The paraffin-embedded tissue was sampled for a polymerase chain reaction (PCR) test to amplify the corresponding pathogen gene sequence, which was *Bartonella henselae*. Therefore, the diagnosis of cat-scratch disease was confirmed. A more detailed medical history showed that, although the patient had no contact history with cats or dogs recently, she was scratched by a dog more than 10 years ago. The patient then received oral rifampicin (450 mg once daily for 4 weeks) and compound sulfamethoxazole (960 mg two times daily for 2 weeks) as part of antibiotic therapy. The follow-up MRI (Figures 1E, F) showed that the residual lesions in the surgical area disappeared at 4 weeks after the operation. No special symptom was found after 6 months of follow-up.

## Discussion

In our study, the case of a 54 year-old woman showed an intracranial CSD involving the dura mater with an imaging appearance similar to that of an atypical meningioma, which has been rarely reported in the literature. In this situation, it is important to make a differential diagnosis between meningioma, solitary fibrous tumor (SFT)/hemangiopericytoma (HPC), and other tumors originating from the dura mater.

Meningioma is one of the most common intracranial tumors originating from the dura mater (7). The clinical manifestation varies greatly, ranging from asymptomatic incidental tumor to fatal tumor. Atypical meningioma is a meningioma subtype with borderline histological and clinical features between benign and malignant meningioma (8). The MRI of atypical meningioma may exhibit a deeper invasion of the dura mater, which is considered an enhancement layer of an asymmetric thickening with manifestations of unclear peritumoral boundary and peritumoral edema (9). After contrast agent administration, atypical meningiomas usually show heterogenous enhancement as the necrosis area may not be enhanced (10). The dural tail on MRI imaging, which is the enhancement of the dura mater involved by the lesion, can be used to distinguish meningioma from other brain lesions. The imaging findings of this case were similar to those of meningioma, such as the dural tail, the peritumoral edema, and inhomogeneous enhancement. Although the patient had no contact history with cats or dogs recently, a more detailed medical history showed that she was scratched by a dog more than 10 years ago. It is difficult to exclude the diagnosis of atypical meningiomas in this case without a biopsy.

Cat-scratch disease may affect almost all organs with a broad range of clinical features and radiological manifestations. The most common clinical features of typical CSD are lymphadenopathy accompanied by constitutional symptoms such as fever, headache, malaise, and nausea (11). In rare cases of atypical CSD, patients may present with atypical clinical manifestations, such as osteomyelitis, discitis, endocarditis, hepatosplenopathy, optic neuritis, and encephalitis (12). Although patients with typical CSD have a history of cat scratching, those with atypical CSD may only have a contact history without scratching, leading to a delayed and difficult diagnosis (3). The mean incubation period of CSD is commonly several weeks. However, in this case, the patient was scratched by a dog more than 10 years ago without a recent contact history with cats or dogs, which may suggest that the incubation period of CSD may be very long.

The clinical features of CSD involving the central nervous system are encephalopathy with headache and altered mental status (4–6). In addition, other neurological manifestations accompanied by encephalopathy may be neuroretinitis, myelopathy, radiculopathy, facial paralysis, cerebral arteritis, and chronic inflammatory demyelinating polyneuropathy (6). The exact pathogenesis of CNS complications is not clear yet. Therefore, various hypotheses have been proposed to explain the mechanism of encephalopathy related to CSD, such as toxic encephalopathy, vasculitis, and direct bacterial invasion of the brain. Whereas, some investigators believe that the possibility of a direct bacterial invasion of the central nervous system is low based on the evidence of the normal cerebrospinal fluid findings and rapid recovery of the patient (4, 13). However, granulomatous inflammation of the brain tissue involving meningitis has been demonstrated by some investigators (14, 15), and the histopathological features of direct infection by *Bartonella henselae* were the same as those in the case of our study. Furthermore, there have been some previous reports of brain abscesses and leptomeningitis related to the infection of *Bartonella henselae* (16–18). Hence, a bacterial invasion may be a continuous process of CSD involving the central nervous system. According to previous reports, CT scans or MRIs of patients with encephalopathy related to CSD may show abnormal findings, including abnormalities in the white matter, the striatum, the thalamus, and the gray matter (19). However, the case of CSD involving the dura mater with dural masses on CT and MRI in our study reflects bacterial infection and the formation of microabscesses and granulations.

In conclusion, CSD is a subacute self-limited infectious disease caused by *Bartonella henselae*, characterized by cat or dog scratching, fever, and subacute regional lymphadenopathy. Its atypical manifestations are diverse, including neuroretinitis, osteomyelitis, and soft tissue mass, which are easy to be misdiagnosed. The case in our study underscores the fact that the incubation period of CSD may be very long. On the contrary, CSD can involve the meninges, resulting in tumor-like lesions.

## Data availability statement

The original contributions presented in the study are included in the article/supplementary material, further inquiries can be directed to the corresponding author.

## Ethics statement

This study was approved by the Ethics Committee of Qilu Hospital of Shandong University. Written informed consent was obtained from the patient for the publication of any potentially identifiable images or data included in this article. The patients/participants provided their written informed consent to participate in this study.

## Author contributions

JH: study concept and design. QF and PW: data analysis. JH: quality assessment. SQ and SL: manuscript. All authors contributed to the article and approved the submitted version.
